# Attitudes towards HPV Vaccination Policy Strategies to Improve Adolescent Vaccination Coverage among Pediatric Providers in New York State

**DOI:** 10.3390/vaccines11081359

**Published:** 2023-08-12

**Authors:** Jana Shaw, Samantha Hanley, Elana Sitnik, Winter Berry, Steven Blatt, Michael Seserman, Margaret K. Formica

**Affiliations:** 1 Department of Pediatrics, Division of Infectious Diseases, Upstate Golisano Children’s Hospital, SUNY Upstate Medical University, Syracuse, NY 13201, USA; 2 Department of Public Health and Preventive Medicine, SUNY Upstate Medical University, Syracuse, NY 13201, USA; hanleysa@upstate.edu (S.H.); sitnike@upstate.edu (E.S.); formicam@upstate.edu (M.K.F.); 3 Department of Pediatrics, Division of General Pediatrics, Upstate Pediatric and Adolescent Center, SUNY Upstate Medical University, Syracuse, NY 13201, USA; berryw@upstate.edu (W.B.); blatts@upstate.edu (S.B.); 4 American Cancer Society, Northeast Region, One Penny Lane, Latham, NY 12110, USA; michael.seserman@cancer.org; 5 Department of Urology, SUNY Upstate Medical University, Syracuse, NY 13201, USA

**Keywords:** human papillomavirus, vaccine, school entry requirement, consent, pharmacy

## Abstract

Pediatric providers’ stances on HPV vaccination-related policies are largely unknown. To gain insight into pediatric providers’ perspectives and potential recommendations for directed policy, we conducted a cross-sectional survey of the American Academy of Pediatrics members in New York. Almost all providers expressed confidence in discussing the HPV vaccine with patients (98.6%, n = 72). Among common barriers to vaccination, providers listed parental safety concerns (n = 60, 82.2%), vaccination not being required for school entry (n = 59, 80.8%), and moral opposition to vaccination (n = 48, 65.8%). Among all respondents, 29 (39.7%), 13 (17.8%), and 2 (2.7%) agreed the vaccine should be required for middle, high, and tertiary school entry, respectively. Support for pharmacist-provision of the vaccine varied, with 31 (42.5%) providers expressing support. Most providers supported adolescent self-consent to vaccination, (n = 67, 91.8%). Providers continued to encounter barriers to HPV vaccination and indicated support of HPV vaccination mandates for school entry, pharmacist provision of the vaccine, and adolescent self-consent to vaccination.

## 1. Introduction

The human papillomavirus (HPV) vaccine was licensed for use in the United States in 2006, and has since demonstrated protection against high-risk oncogenic strains of the virus [1]. The Centers for Disease Control and Prevention (CDC) recommends that both females and males receive the vaccine at the age of 11 or 12 years. Timely vaccination is important, as the vaccine works best when administered before HPV exposure and requires fewer doses when given prior to age 15 [2]. In Scotland, routine vaccinations with the bivalent HPV vaccine of girls aged 12–13 years led to a significant reduction in early-stage cervical diseases. Compared with unvaccinated women, those who received the vaccine experienced a remarkable 89% decrease (with a 95% confidence interval between 81% and 94%) in cervical intraepithelial neoplasia (CIN) grade 3 or worse. Additionally, there was an 88% reduction (ranging from 83% to 92%) in CIN grade 2 or worse, and a 79% reduction (ranging from 69% to 86%) in CIN grade 1. The effectiveness of the vaccine was found to be greater in those vaccinated at a younger age. Women who received the vaccine at ages 12–13 showed an 86% reduction (ranging from 75% to 92%) in CIN grade 3 or worse, while those vaccinated at age 17 had a lower effectiveness of 51% reduction (ranging from 28% to 66 percent) [3]. Similar findings showing the benefits of HPV vaccination in cervical cancer prevention were reported in the USA, Australia, and Sweden [4,5,6].

Despite this high vaccine effectiveness and safety, national percentages among adolescents aged 13–17 years show that only 61.7% of adolescents were up to date with HPV vaccinations in 2021, with coverage increasing slightly from 58.6% in 2020; overall, only 76.9% of adolescents received at least one HPV vaccine dose in 2021, a modest increase of 1.8% from 2020 [2]. Among New York State (NYS) adolescents aged 13–17 years, HPV vaccination completion rates also remain low at 64.4%, and are even lower for vaccine series completion by 13 years of age, at 40.2% [7,8]. These rates fall far below the 2030 Healthy People target coverage goal of 80%, representing a missed opportunity to protect all children in NYS from HPV-related cancer [9].

HPV vaccination rates have historically lagged behind coverage for other adolescent vaccines, and the coronavirus disease (COVID-19) pandemic led to delayed routine childhood vaccine delivery [2,10]. Efforts to improve HPV vaccination coverage have included the implementation of evidence-based practices and interventions, such as provider education and incentives, patient education, outreach and scheduling, systems-level operational changes in clinical practice settings such as electronic health record alerts and reminders, and the use of multidisciplinary approaches [11].

Unfortunately, current interventions only modestly increase vaccination coverage and require frequent reinforcement. Additional multi-faceted approaches related to HPV vaccine access and uptake are needed to increase HPV vaccination rates. Potential strategies include HPV vaccination requirements for school entry, the expansion of adolescent consent for vaccination, and pharmacist-administered vaccination.

Numerous research studies have consistently shown that regions with school-entry vaccination requirements for general vaccines tend to have higher immunization coverage compared to areas without such mandates. Recent data also indicate that the positive impact of these requirements extends to the HPV vaccine as well. A pre-post design study focused on the HPV school-entry policy in Puerto Rico revealed compelling evidence of improved HPV vaccination rates following the implementation of the school-entry policy. A significant rise in the initiation of the HPV vaccine was observed among 11–12-year-old adolescents in the years 2017 (a pre-policy year), 2018, and 2019, with rates of 58.3%, 76.3%, and 89.8%, respectively. Furthermore, there was a notable reduction in the gap between males and females regarding vaccine initiation and up-to-date coverage over time [12]. In a separate cross-sectional study conducted in three U.S. jurisdictions with HPV immunization school-entry requirements, notably higher levels of vaccination initiation were observed compared to regions without such policies within the same area. Specifically, when comparing policy and non-policy jurisdictions in the same region, all jurisdictions implementing the policy, except for Virginia, demonstrated significantly greater changes in vaccination initiation rates for both girls and boys before and after the policy’s implementation. In the District of Columbia, the mean post-policy HPV vaccination initiation rates showed substantial increases for girls aged from 13 to 15 years (from 33.0% to 67.2%) and for those aged from 16 to 17 years (from 41.7% to 74.5%), respectively. Similarly, for boys aged from 13 to 15 years, the mean post-policy HPV vaccination initiation rates increased from 37.6% to 76.7%, and for boys aged from 16 to 17 years, initiation rates increased from 47.2% to 85.6%, respectively. Following the introduction of the HPV vaccination school-entry policy in Rhode Island, there were noteworthy improvements in mean vaccination initiation rates for girls aged from 13 to 15 years (from 68.6% to 90.9%) and for those aged from 16 to 17 years (from 80.8% to 86.5%), respectively [13]. These findings indicate the positive impact of school-entry requirements on increasing HPV vaccination rates across different age groups for boys and girls. The perspective of NY pediatric providers regarding the school-entry HPV immunization requirement is currently unknown.

As adolescents grow older, they may desire more autonomy and involvement in decisions about their own healthcare, including vaccinations. This desire for autonomy may be incompatible with the legal requirements that mandate parental consent for vaccinations. It has been previously shown that adolescents often feel excluded from the decision-making process concerning their vaccination, despite their strong desire to be involved in such decisions [14]. A major hindrance to vaccination among adolescents is the requirement in most states, including New York State (NYS), that individuals must be 18 years or older to give consent to vaccination. Few exemptions exist for emancipated minors or sexually active adolescents. In NYS, healthcare providers are authorized to provide medical care for sexually transmitted diseases to minors, including the administration of HPV vaccination to sexually active adolescents, without requiring consent from their parents or guardians. However, this situation presents a potential concern: given that the decision to receive the HPV vaccine may imply that the teen is sexually active, some adolescents may hesitate to receive vaccination due to the fear of facing negative reactions or repercussions from their parents. This fear of parental backlash may act as a barrier to their willingness to get vaccinated, even though it is crucial for their health and protection against HPV-related risks. Currently, health care providers’ perspective on the extension of the rights of adolescents to consent to HPV vaccination by eliminating the stipulation of sexual activity is unknown.

Pharmacists also play an essential role in vaccination by enhancing access to immunization services, promoting vaccine education, and improving vaccination rates in communities. The involvement of pharmacists in childhood influenza vaccination has been a significant development in public health, increasing vaccination rates and improving access to preventive care for children. Their expanded role has helped make the influenza vaccine more convenient and accessible to families, ultimately contributing to the overall efforts to prevent and control seasonal influenza [15]. According to a 2018 online survey of US physicians, family medicine physicians were more likely than pediatricians to support the administration of HPV vaccination by trained pharmacists (adjusted odds ratio [OR] = 1.62, 95% confidence interval [CI] [1.17–2.22]). In a 2020 news release, the American Academy of Pediatrics expressed its opposition to the provision of childhood vaccines by pharmacies, stating: “Creating a new vaccine system is not only unnecessary, but it will not provide children with the same level of optimal medical care they receive from the pediatrician who knows the child’s medical history” [16]. The support for pharmacist provision of the HPV vaccine among pediatric healthcare providers in New York remains unknown.

In order for HPV-related policies to be successful, several conditions need to be met, including, although not limited to vaccine coverage and funding through the Vaccines For Children (VFC) program, public acceptance of vaccine efficacy and safety, the addition of the vaccine to immunization information systems, and physician/provider support for the vaccine, and vaccine mandates. A thorough evaluation of vaccine mandates is essential, considering various aspects, such as epidemiological, economic, and ethical concerns. It is crucial to approach the implementation of mandates with care, as any inappropriate application may lead to a loss of support for immunization programs and undermine the progress achieved through existing policies and initiatives [17]. There is a gap in the understanding of the perspectives of pediatric providers on policies related to pharmacy provision of the HPV vaccine, school entry immunization requirements, and minor consent. To address this gap, we conducted a survey of American Academy of Pediatrics (AAP) members in New York State to obtain insights into their perspectives on these policies.

## 2. Methods

### 2.1. Study Design

This research study aims to investigate the attitudes and beliefs of pediatric healthcare providers regarding HPV-related policies. Our study adopted a cross-sectional survey design to gather data from a sample of pediatric providers in New York State. By examining providers’ perspectives on HPV vaccination policies, we sought to gain insight into potential barriers, facilitators, and recommendations for enhancing vaccination rates and policy implementation.

### 2.2. Study Instrument

Our survey instrument was carefully developed using validated questions from previously published research exploring provider’s attitudes, beliefs, and HPV-vaccine-related practices [18,19,20]. Each question underwent a thorough and rigorous review process to ensure their relevance, comprehensiveness, and alignment with the research objectives. To guarantee the clarity of each question, the survey was pilot-tested by all authors independently and adjusted as needed. A final 29-item survey containing Likert-scale and multiple-choice questions was divided into the following sections: provider demographic information, current HPV vaccination practices, HPV vaccination policy attitudes and beliefs, and recommendations for policy improvement.

*Provider Demographic Information:* Standard demographic data were collected, including information on participant gender, race, ethnicity, practice type and location, role, years of experience in clinical practice, and practice setting. We also gathered information on HPV vaccine acceptance, as well as the uptake of other childhood vaccines, in the past year.

*Current HPV Vaccination Practices:* Likert-scale questions were used to determine current vaccination practices, including the level of provider recommendations for adolescents. This section also sought to identify perceived barriers to HPV vaccination (such as lack of reimbursement, vaccine administration, and time spent on risk communication/patient education).

*HPV Vaccination Policy Attitudes and Beliefs:* Likert-scale and multiple-choice questions were used to gauge provider perception of potential HPV vaccination policies, including their level of agreement and support for different policy components. Specifically, questions about HPV vaccine school-entry requirement, pharmacist provision of HPV vaccine, and expansion of adolescent consent were included.

*Recommendations for Policy Improvement*: We elicited additional suggestions and/or recommendations for improving HPV vaccination-related policies by including an open-ended question. We aimed to explore new themes or issues that we might not have considered before. This allowed participants to bring up topics that are important to them, even if those topics were not initially included in the survey, and to allow respondents to answer in their own words, reducing the risk of their responses being led or influenced.

### 2.3. Participants and Recruitment

Members across all three chapters of the New York State American Academy of Pediatrics (AAP) were invited via email to participate in the online survey between June and August 2021. The survey was open to pediatricians, family medicine physicians, resident physicians, nurse practitioners, physician’s assistants, and administrators, as well as other members of AAP. Members received a survey invitation e-mail, which included a detailed description of the study’s objectives as well as a link to the survey. The survey was voluntary, anonymous, and not incentivized. The voluntary nature of participation was communicated in the introductory invitation email and reiterated at the beginning of the survey to confirm informed consent. Participant identities were also completely anonymized and protected throughout the study to ensure confidentiality. Two e-mail reminders were sent during the survey progress to encourage participation.

### 2.4. Data Collection

Study data were collected and managed using Research Electronic Data Capture (REDCap) tools hosted by the State University of New York (SUNY) Upstate Medical University [21]. The research protocol was reviewed and approved by the SUNY Upstate Institutional Review Board, and it was determined that the project was exempt from IRB review, project #1759421-1.

### 2.5. Statistical Analysis

By employing a combination of quantitative and qualitative data analysis techniques, we aimed to provide a comprehensive and well-rounded assessment of the survey outcomes. These analytical approaches enabled us to draw meaningful conclusions, identify significant patterns, and derive valuable insights that will serve as the foundation for informed decision-making and further research.

The data analysis process involved an examination of quantitative data obtained from Likert-scale and multiple-choice questions. Descriptive statistics, such as frequencies and percentages, were employed to summarize and present the responses in a clear and concise manner. The Likert-scale responses were organized into five categories: “Strongly Agree,” “Agree,” “Not Sure,” “Disagree,” and “Strongly Disagree.” To simplify the interpretation of results and facilitate meaningful comparisons, we collapsed the five categories into three broader ones: “Agree,” “Neutral,” and “Disagree.” This grouping allowed us to gain a more comprehensive understanding of the general sentiment towards the survey questions. To investigate potential relationships between survey responses and specific provider and practice characteristics, we utilized chi-square statistics. This statistical method enabled us to assess the association between categorical variables, providing valuable insights into how certain factors might influence participants’ opinions and perceptions.

Moreover, qualitative data obtained from open-ended questions allowed respondents to express their thoughts freely and in their own words. To analyze this qualitative data, we employed content analysis, a systematic approach that involved carefully examining and categorizing the responses to identify recurring themes. This qualitative analysis complemented the quantitative findings, offering deeper insights and a contextual understanding of the survey results.

The statistical analysis process was conducted using SAS v. 9.4 (Cary, NC), a widely recognized and robust software package for data management and statistical analysis. SAS’s powerful capabilities allowed us to perform various complex calculations and ensure the accuracy and reliability of our findings.

## 3. Results

### 3.1. Socio-Demographic and Clinical Practice Characteristics

During the survey period, 4353 members of AAP were invited via email to participate in the online survey. A total of 82 providers responded to the survey. Among the respondents, 9 providers were excluded because they did not answer any vaccine-related survey questions, leaving 73 respondents for the final analysis. The majority of the respondents were females, n = 47 (64.4%), and self-reported as non-Hispanic White, n = 61 (83.6%). The remainder of respondents’ socio-demographic characteristics were as follows: 3 (4.1%) were non-Hispanic Black, 5 (6.8%) were Asian, 1 (1.4%) were Hispanic, and 3 (4.1%) identified as “other” or nondisclosed by choice. Most survey respondents, n = 60 (82.2%), were pediatricians and over half of respondents, n = 42 (57.5%), had more than 20 years of clinical experience in practice. The majority of providers, n = 41 (56.2%), saw between 10 and 24 adolescent patients per week on average, as shown in Table 1.

Among respondents, 54 (73.9%) spent 11 or more years in clinical practice, 48 (65.8%) saw 50–100 patients a week on average, 34 (46.6%) of providers identified their practice type as private, independent, 23 (31.5%) hospital or medical center, 10 (13.7%) community health center or federally qualified health center (FQHC), and 6 (8.2%) indicated “other.” Half of the respondents worked in a suburban location, 37 (50.7%), while 24 (32.9%) reported urban, and 12 (16.4%) reported rural settings. Percentage of patient participation in the VFC varied, as 13 (17.8%), 25 (34.3%), 20 (27.4%) respondents reported <25%, 25–50%, >75%, of their patients participated in VFC, respectively (Table 1). The proportion of respondents reporting parents refusing the HPV vaccine, on average, varied between <10% and ≥50%; however, most providers, n = 61 (83.6%) indicated that less than 10% of parents refused other recommended childhood vaccines (Table 1).

### 3.2. Members’ Experience with HPV Vaccination

Almost all providers, n = 72 (98.6%), expressed confidence in discussing the HPV vaccine with patients, and reported recommending the vaccine to parents of all eligible adolescents, n = 71 (97.3%). Among common barriers to vaccination, providers listed parental safety concerns, n = 60 (82.2%), vaccine not being required for school entry, n = 59 (80.8%), and moral opposition to vaccination, n = 48 (65.8%). Almost half of respondents also identified parental concern that the vaccine encourages early initiation of sexual activity as a barrier to vaccination coverage, n = 35 (48%). Very few providers, n = 7 (9.6%) agreed that lack of reimbursement for the vaccine, vaccine administration or time spent on risk communication and/or patient education are barriers to vaccination (Table 2).

### 3.3. Members’ Attitude toward School Entry HPV Immunization Requirement

Among all respondents, 29 (39.7%), 13 (17.8%), and 2 (2.7%) agreed the vaccine should be required for middle, high, and tertiary school entry, respectively. One-third of providers indicated they do not believe the HPV vaccine should be required for any level of school entry, n = 24 (32.9%) (Table 3). Female providers were more likely than male providers to support school entry mandates (66.0% vs. 42.3%, *p* = 0.05). Providers working in suburban locations were also more likely to agree that the vaccine should be mandated for school entry, compared to those working in urban and rural settings (73.0% vs. 50.0% vs. 25.0%, *p* = 0.009) 

### 3.4. Members’ Perspective on Pharmacist Provision of HPV Vaccine

Support for pharmacist provision of the vaccine varied with 31 (42.5%), 14 (19.2%), 28 (38.4%) providers expressing support, neutrality or no support for HPV vaccination, respectively (Table 3). Among those who indicated *support* for pharmacist provision (n = 31), most respondents believed that pharmacies provide a convenient point of access for patients, n = 30 (96.8%); pharmacists offer extended hours of operation, during which patients can be vaccinated, n = 25 (80.6%); pharmacists are required to enter vaccine administration into the immunization registry, n = 23 (74.2%); pharmacists have adequate experience and training to provide vaccine, n = 20 (64.5%); and pharmacists have adequate experience to provide counseling, n = 13 (41.9%). The proportion of providers who agreed with pharmacist provision of the vaccine was inversely correlated with the number of adolescent patients seen per week (<10: 68.8%, 10–24: 36.6%, ≥25: 31.3%, *p* = 0.05).

Among those who indicated *opposition* to pharmacist provision of the vaccine (n = 28), all expressed wanting to ensure continuity of care for patients, and most believed their practice would provide a safer vaccination, n = 20 (71.4%). In addition, 19 (67.9%) respondents were concerned that pharmacists providing the vaccine do not know the patient and their medical history,12 (42.9%) respondents did not believe pharmacists have adequate experience vaccinating children and adolescents, 10 (35.7%) did not believe that pharmacies were prepared to handle vaccine-related adverse reactions, and 9 (32.1%) respondents were concerned about loss of revenue.

Among those who expressed a *neutral* position on pharmacist provision of HPV vaccination, n = 14 (19%), 11 (78.6%) wanted to ensure continuity of care for their patients, 6 each (42.9%) did not believe pharmacies were prepared to handle vaccine-related reactions, and were concerned that pharmacists providing the vaccine did not know the patient and their medical history, respectively. Five (35.7%) respondents were concerned about loss of revenue, and 4 (36.4%) did not believe pharmacists had adequate experience vaccinating children and adolescents.

### 3.5. Members’ Perspective towards Adolescent Consent to HPV Vaccination

Regarding consent, most providers, n = 67 (91.8%) indicated support for adolescent self-consent to vaccination, regardless of risk of a sexually transmitted infection, 4 (5.5%) expressed a neutral position, and 2 (2.7%) expressed strong disagreement with the right of adolescents to self-consent to HPV vaccination (Table 3). Pediatricians were more likely than other healthcare providers to agree with adolescent self-consent for the vaccine (95.0% versus 76.9%, *p* = 0.03). While only two providers indicated that they disagreed with the right for adolescents to self-consent for the HPV vaccine, both providers did agree on two reasons for their opinions. Both providers specified that they felt adolescents may not be mature enough to make decisions that impact their health. Both providers also indicated that they believe that adolescents’ patients lack the capacity to make informed decisions regarding vaccination. Interestingly, one of the two providers also commented that adolescence encompasses a broad age range, so self-consent for the HPV vaccine may not be appropriate for younger adolescents, while it may be appropriate for older adolescents.

## 4. Discussion

In this survey, of a sample of AAP members in NYS, we found that providers continue to encounter previously identified barriers to HPV vaccination (i.e., parental safety concerns, vaccines not being required for school entry, moral opposition to vaccination, and concern that the vaccine encourages early initiation of sexual activity) despite overwhelming scientific evidence indicating otherwise [22,23,24]. This is consistent with recent findings showing a 10.4 percentage point increase in adolescent caregivers declining HPV vaccination due to vaccine safety concerns as the primary reason for HPV vaccination refusal, 13.0% (95% CI, 12.1–14.0%) in 2015 to 23.4% (95% CI, 21.8–25.0%) in 2018, respectively (*p* < 0.001) [25].

More than half (60.2%) of responding providers indicated support for HPV vaccination mandates for middle school, high school, or college entry as a strategy to increase HPV vaccination rates. Respondents working in suburban locations were more likely to support vaccination mandates compared to those working in urban and rural settings. Extant research suggests that geographic disparities in vaccination uptake exist. Adolescents living outside of metropolitan statistical areas (MSAs), as defined by the U.S. Census Bureau, have lower coverage of childhood vaccinations. The coverage disparity between MSA and non-MSA geographic regions is the largest for HPV vaccination. Notably, adolescents living below the federal poverty level have higher rates of HPV vaccine coverage, regardless of geographic location, which may be attributable to access to the VFC program [26]. Further research is needed to assess the relationship between geographic location, socioeconomic status, and vaccine barriers. However, we propose these findings may be attributed to the greater burden of parental vaccine hesitancy occurring outside of MSA regions, which may explain increased AAP member support of HPV vaccination mandates in suburban NYS. Vaccination requirements for school entry may prove to be a great equalizer, as mandates encourage a standardized age of vaccine administration and facilitate the even distribution of information regarding the benefits of vaccination. Research shows that states with HPV vaccination requirements for school entry experience increased vaccination initiation among adolescents aged from 13 to 17 years [13]. Therefore, we believe that the adoption of school-entry requirements of HPV vaccination should be given strong consideration in NYS to increase vaccine uptake and ensure that all children are given the opportunity to benefit from HPV vaccination and, ultimately, cancer prevention.

Almost half of respondents (42.5%) support pharmacist provision of the HPV vaccine. Historically, pharmacists have played a key role in providing vaccinations to adults and children during national health emergencies such as the 2009 H1N1 influenza and the COVID-19 pandemics [26,27]. In NYS, pharmacists were authorized to administer seasonal influenza vaccines to children between 2 and 18 years of age, and to also provide HPV vaccinations under the Public Readiness and Emergency Preparedness Act [28,29]. The White House Administration ended national and public health emergencies on 11 May 2023, however, which ended this benefit to children and their families [30]. However, pharmacists are some of the most accessible health care providers within the community, frequently interacting with patients and their families, which uniquely positions them as a trusted source for vaccine administration. The accessibility, hours of operation, and convenient location of most pharmacies may reduce barriers to vaccine uptake, especially for those unable to regularly access their primary health care provider during standard business hours. We believe that expanded pharmacist authorization to provide HPV vaccination has strong potential to increase vaccine coverage, as adolescents and their families may also be able to access information and vaccination more readily than they would from their primary care provider.

An overwhelming majority (91.8%) of respondents were also in support of allowing adolescents to consent to HPV vaccination, regardless of their sexual behavior. Currently, health care providers in NYS are permitted to provide HPV vaccinations to sexually active minors without parental/guardian consent [31]. However, the law does not ensure the confidentiality of minors, since HPV vaccination records are publicly available via the NYS Immunization Information System, as well as through health plan statements. Given the availability of this information, acceptance of the vaccine may signal adolescent sexual activity to parents. Establishing the legal right of minors to consent to vaccination, regardless of sexual behavior or intent, empowers health care providers to independently assess patient maturity and capacity to choose HPV vaccination, and effectively navigate family dynamics in the interests of patients whose parents oppose vaccination. We have previously argued that, in health care, both law and ethics have long recognized the rights of adolescents under age 18 to make their own independent decisions based on status (e.g., married, emancipated) or the nature of their health care concerns, including decisions about testing and treatment for sexually transmitted infections, reproductive health, substance abuse and outpatient mental health [32]. Independent adolescent consent to HPV vaccination should be an ethical and policy priority without the stigma of known or anticipated sexual activity, as dictated by current practice.

This study had several limitations. The low response rate to the survey (estimated at 2% based on 4353 AAP registered members during the study period), primarily due to the survey timing, may have introduced selection bias, as those who chose to participate may have different perspectives or experiences related to HPV vaccination compared to non-respondents. This could limit the generalizability of the findings to the broader population of New York State AAP members. Providers were surveyed during the COVID-19 pandemic, which generated provider fatigue and time constraints over competing priorities, especially for online content such as telehealth visits, webinars, meetings, and surveys. In addition, financial constraints on pediatric offices during the pandemic have been significant, possibly creating a sentiment of there being little allowable time or interest in non-incentivized survey requests. The study relied on self-reported responses from participants, which introduced the possibility of response bias. Providers’ answers might have been influenced by social desirability bias or recall bias, potentially leading to the over- or underrepresentation of certain attitudes or practices related to HPV vaccination. The accuracy of the responses may also be influenced by subjective interpretation or understanding of the survey questions.

Although we had limited information regarding AAP member demographics, we were able to confirm a proportion of male to female respondents that reflected the population being surveyed. Survey respondents were 35.6% male and 64.4% female, which is similar to the AAP members (32.3% male and 67.0% female). In addition, while almost half of AAP members do not disclose race or ethnicity, among those who do, the race/ethnicity distribution is similar to our survey respondents. Our survey was cross-sectional, and therefore represents respondents’ perspective at the time of survey. Longitudinal studies would provide a more comprehensive understanding of how provider attitudes and practices regarding HPV vaccination policy may evolve. The study focused specifically on New York State AAP members, which may limit the generalizability of the findings to other regions or healthcare providers. The study primarily targeted pediatric providers, and the perspectives of other healthcare professionals involved in HPV vaccination, such as family practitioners or gynecologists, were not captured. Future research should focus on validation of these findings among the national AAP membership base, as well as expanding the present knowledge and understanding of HPV policy views among other vaccinating health care workers in non-pediatric offices, community health clinics, school-based health centers, and health departments.

## Figures and Tables

**Table 1:** Members’ sociodemographic and practice characteristics (N = 73).

Sociodemographic Characteristics	N	%
Gender		
Male	26	35.6
Female	47	64.4
Race/Ethnicity		
White	61	83.6
Black or African American	3	4.1
Asian	5	6.8
Hispanic/Latinx Origin	1	1.4
Other/Prefer Not to Disclose	3	4.1
Role		
Pediatrician	60	82.2
Other	13	17.8
Years in Clinical Practice		
<5	12	16.4
5–10	7	9.6
11–20	12	16.4
>20	42	57.5
Number of Patients Per Week		
<50	20	27.4
50–100	48	65.8
>100	5	6.8
Number of Adolescent Patients Per Week		
<10	16	21.9
10–24	41	56.2
>24	16	21.9
**Practice Characteristics**		
Type		
Private, Independent Practice	34	46.6
Hospital/Medical Center/Practice Network	23	31.5
Community Health Center/FQHC	10	13.7
Other	6	8.2
Location		
Urban	24	32.9
Suburban	37	50.7
Rural	12	16.4
Percentage of Patients that Participate in VFC		
<25%	13	17.8
25–50%	25	34.3
51–75%	8	11.0
>75%	20	27.4
Practice Does Not Participate	3	4.1
Unsure	4	5.5
Percentage of Patients that Refuse HPV Vaccine		
<10%	23	31.5
10–19%	18	24.7
20–29%	18	24.7
30–49%	8	11.0
≥50%	4	5.5
Unsure	2	2.7
Percentage of Patients that Refuse Other Childhood Vaccines		
<10%	61	83.6
10–19%	5	6.9
20–29%	3	4.1
30–49%	0	0.0
≥50%	2	2.7
Unsure	2	2.7

**Table 2:** Respondents’ current HPV vaccination practices and reported barriers to providing HPV vaccination (N = 73).

Agree or Strongly Agree	N	%
Lack of reimbursement for the vaccine, vaccine administration, or time spent on risk communication/patient education	7	9.6
Up-front costs of purchasing, storing, and providing the vaccine.	2	2.7
Additional time it takes to talk about the vaccine.	23	31.5
Adding another vaccine to the vaccine schedule.	10	13.7
Difficulty ensuring 2 or 3 doses will be completed.	12	16.4
Obtaining parental consent when a parent or legal guardian is not present.	14	19.2
Vaccine is not required for school entry.	59	80.8
Parent concern about the safety of HPV vaccine.	60	82.2
Patient concern about the safety of HPV vaccine.	16	21.9
Parent concern about the safety of efficacy of HPV vaccine.	12	16.4
Patient concern about the safety of efficacy of HPV vaccine.	6	8.2
Parent moral opposition to the HPV vaccine.	48	65.8
Patient moral opposition to the HPV vaccine.	13	17.8
I feel confident discussing the HPV vaccine with my patients.	72	98.6
I discuss HPV vaccination with parents only if they ask about the vaccine.	6	8.2
I discuss HPV vaccination with all of my eligible patients.	69	84.5
I recommend the HPV vaccine to the parents of all eligible adolescents.	71	97.3
I am confident I can address specific parental concerns and questions about HPV.	65	89.0
I am confident that I can address the concern that HPV vaccination may increase adolescent sexual activity.	71	97.3
I have enough time during visits to probe parents about their reasons for wanting to refuse or delay HPV vaccination.	35	48.0
I am influential in parents’ final decision about whether to get the HPV vaccine for their child.	46	63.0
I am usually able to convince hesitant parents to get the HPV vaccine.	33	45.2
When parents wish to delay or refuse HPV vaccination, there is not much I can say to change their minds.	23	31.5
I always review vaccination status with adolescent patients during annual well visits.	67	91.8
I strongly recommend on-time HPV vaccination for girls.	58	79.5
I strongly recommend on-time HPV vaccination for boys.	64	87.7

**Table 3:** Respondents’ perspective toward vaccine mandates, pharmacist provision, and adolescent self-consent (N = 73).

Perspective	N	%
Believe that the HPV vaccine should be required for school entry.		
Yes, for middle school entry and beyond	29	39.7
Yes, but for high school entry and beyond	13	17.8
Yes, but for college entry and beyond	2	2.7
No, do not believe HPV vaccine should be required	24	32.9
No opinion	5	6.9
Support pharmacist provision of the HPV vaccine for adolescents.		
Agree/Strongly Agree	31	42.5
Neutral	14	19.2
Disagree/Strongly Disagree	28	38.4
Support the right of adolescents to self-consent to HPV vaccination.		
Agree/Strongly Agree	67	91.8
Neutral	4	5.5
Disagree/Strongly Disagree	2	2.7

## Data Availability

Data can be provided upon request to the corresponding author.
